# Family resilience and adaptive coping in children with juvenile idiopathic arthritis: protocol for a systematic review

**DOI:** 10.1186/s13643-017-0619-z

**Published:** 2017-11-02

**Authors:** Sophia Saetes, Lisa Hynes, Brian E. McGuire, Line Caes

**Affiliations:** 1 School of Psychology & Centre for Pain Research, National University of Ireland, Galway, Ireland; 2 Department of Psychology, West Virginia University, Morgantown, WV USA; 3 School of Natural Sciences, University of Stirling, Stirling, Scotland UK

## Abstract

**Background:**

This systematic review is the first step in a study investigating the resilience methods and processes in families of children with juvenile idiopathic arthritis. In particular, this review will focus on chronic or persistent pain, as a common symptom of juvenile idiopathic arthritis, which is the most common rheumatic disease in childhood. The experience of persistent pain can add to the functional disability associated with juvenile idiopathic arthritis. Resilience has relevance to all areas of paediatric psychology, and targeted attention to child, sibling, and parent strengths within the context of paediatric chronic pain and juvenile idiopathic arthritis in particular will augment the field on numerous levels. The objective is to determine which resilience processes are associated with a favourable quality of life in terms of academic, communication, emotional, interpersonal, physical, psychological, and social well-being in families of children with chronic pain associated with JIA.

**Methods/design:**

This systematic review will be conducted and reported in accordance with the Preferred Reporting Items for Systematic Reviews and Meta-Analyses statement and the PRESS (Peer Review of Electronic Search Strategies) guideline.

Longitudinal, cross-sectional, and treatment studies written in English will be included, as will grey literature (i.e. conference abstracts and dissertations).

Studies involving participants who are 6–18 years of age, have been diagnosed with juvenile idiopathic arthritis, are experiencing chronic pain, and are currently undergoing treatment will be included regardless of sex, arthritis type, and type of treatment. Studies including siblings who are 6–18 years of age and the patient’s parents will be included.

**Discussion:**

Research exploring resilience within the adult population is accruing. Shifting our focus to protective factors of resilience in the context of paediatric chronic pain, specifically juvenile idiopathic arthritis, is a novel and promising pursuit that has the potential to optimize and inform future clinical practice and interventions. A better understanding of the role of reliance in family adaptation will facilitate the development of more effective treatment approaches and lay the foundation for more effective self-management in paediatric chronic pain.

**Systematic review registration:**

This protocol is registered with the International Prospective Register of Systematic Reviews (PROSPERO) database (registration: CRD42016047226).

**Electronic supplementary material:**

The online version of this article (10.1186/s13643-017-0619-z) contains supplementary material, which is available to authorized users.

## Background

### Description of the condition

This systematic review is the first step in a larger study investigating the resilience methods and processes in families of children with juvenile idiopathic arthritis (JIA). In particular, this review will focus on chronic or persistent pain, a common symptom of JIA, which is the most common rheumatic disease in childhood. Symptoms of JIA include joint pain and stiffness, fevers, rash, bone infection, and inflammation. JIA frequently continues into adulthood and can end in substantial long-term morbidity, including physical disability [1, 2]. The experience of persistent pain can add to the functional disability associated with JIA.

### Why is resilience important?

In the context of paediatric traumatic chronic illness such as JIA, resilience may be a one process that determines whether the child and their family experience traumatic stress or post-traumatic growth. Resilience is considered a protective factor and can be defined as “a dynamic and multi-systemic progression that allows the individual to respond effectively when faced with risk or adversity (e.g. medical condition)” [3]. The process of resilience originates within the individual and is enhanced through developmental, social, cultural, and environmental factors. Resilience encompasses the concepts of posttraumatic growth, benefit-finding, optimism, and hope. Only a few studies have focused on these concepts in children and adolescents outside the context of childhood cancer, but preliminary evidence suggests that these constructs may be related to good adjustment in various paediatric chronic illnesses [4]. Current practices pertaining to paediatric chronic pain are guided largely by an adult-focused body of research, which is unsuccessful in taking into account relevant developmental factors such as cognitive and social development and complex familial factors [5]. Further research into the presence, assessment, and utilization of resilience within paediatric chronic pain is necessary.

Resilience has relevance to all areas of pediatric psychology, and targeted attention to child, sibling, and family strengths within the context of paediatric chronic pain and JIA in particular will augment the field on numerous levels [6]. For instance, by refining the particular strengths or resilience resources that are of relevance for adaptive adjustment to childhood JIA, these processes can be targeted in clinical practice in order to enhance psychological, cognitive, and social outcomes for families of children with JIA.

While there are several models emerging in the field that aid in the measurement of resilience, these models require more research on how they may apply to resilience theory. Furthermore, though resilience theory includes risk exposure within adolescents, it primarily concentrates on healthy growth despite risk exposure. As such, the authors chose to engage in a broad approach (the inclusion of both resilience resources and mechanisms) for this systematic review.

## Objective

The objective of this study is to determine which resilience processes are associated with a favourable quality of life, in terms of academic, communication, emotional, interpersonal, physical, psychological, and social well-being, in families of children with chronic pain associated with JIA.

## Methods

### Criteria for considering studies for this review

This systematic review, which will result in a narrative synthesis of the current evidence on family resilience in JIA, will be conducted and reported in accordance with the Preferred Reporting Items for Systematic Reviews and Meta- Analyses (PRISMA) statement and the PRESS (Peer Review of Electronic Search Strategies) guideline. The PRISMA-P+ checklist document shows this in more detail [see Additional file 1] [7, 8]. The PRISMA-P checklist encourages authors to describe eligibility criteria using the PICO reporting system (which describes the participants, interventions, comparisons, outcome(s), and study design of the included studies) [8]. This systematic review protocol is registered with the International Prospective Register of Systematic Reviews (PROSPERO) database (registration number: CRD42016047226).

### Types of studies

Longitudinal, cross-sectional, and treatment studies written in English will be included. There were no limitations with respect to the design of treatment studies to be eligible for inclusion. Grey literature (i.e. conference abstracts and dissertations) will be included. Review articles will be excluded from the search, as will studies for which the full text is not available.

### Types of participants

Studies involving young people who are 6–18 years of age, who have been diagnosed with JIA, who are experiencing chronic pain, and who are currently undergoing treatment will be included regardless of sex, arthritis type, and type of treatment. Studies including siblings who are 6–18 years of age and the patient’s parents will also be included.

## Types of outcome measures

### Primary outcomes

Included studies must have a variable reflecting resilience as either the primary outcome of interest or as a key predictor, moderator, or mediator. This will include resilience utilized as a resource or a mechanism. Resilience resources can include optimism, social and community support, self-esteem, problem-solving/active coping, positive family/social interactions, and adaptive family functioning [9]. Resilience mechanisms identified specifically for the context of pain are pain acceptance, pain-related self-efficacy, parental acceptance of pain, and parent psychological flexibility [9]. Resilience has been operationalized by several terms, including but not limited to post traumatic growth or benefit seeking, adjustment or adaptation, self-esteem, self-concept, optimism, and hope, all of which will be searched for.

### Secondary outcomes

Studies evaluating the impact of resilience variables, either direct or indirect, on children with JIA and their families in terms of impact on quality of life and functional outcomes (academic, communication, emotional, interpersonal, physical, psychological, and social) will be included.

## Search methods for identification of studies

No date restriction will be imposed on the studies. Studies will be included if a full-text paper in English is available, either through databases or through contact with the study authors. Where available, protocol methods will be compared with the methods and results reported in the included study.

### Electronic searches

The following electronic databases will be searched: Web of Science, MEDLINE (Ovid), EMBASE, Psychology and Behavioral Sciences Collection (EBSCO), Psycarticles (Ovid), and PsycINFO (Ovid). This can be seen in Table 1. The same search strategies will be used with alterations as appropriate for each database interface.

**Table 1:** Electronic searches

Search strategy
1. (Juvenile Idiopathic Arthritis or JIA or rheumatoid arthritis or systemic-onset Juvenile Idiopathic Arthritis or psoriatic arthritis or enthesitis-related Juvenile Idiopathic Arthritis or oligoarthritis or polyarthritis).
2. AND (Chronic pain or recurrent pain or pain).
3. AND (Children or child or adolescence or adolescent or pre adolescence or pediatric or paediatric).
4. AND (Sibling or family or family function or parent or parenting or parental or peer relationships).
5. AND (Resilience or resiliency or post traumatic growth or optimism or benefit seeking or benefit finding or coping skills or coping or adjustment or adaptation or health behavior or health behaviour or quality of life or hope or psychological resilience or psychosocial functioning or social support or self-concept or acceptance or self-efficacy or positive affect).

### Searching other resources

Unpublished and ongoing trials will be identified by checking trials and protocols published on relevant databases of current ongoing clinical research studies (e.g. https://clinicaltrials.gov). Trial registries will also be searched. The lead or contact authors of all identified studies will be asked to identify further studies where possible. Grey literature will be searched using the Open Grey database (www.opengrey.eu/), which includes technical or research reports, doctoral dissertations, and conference papers from the previous 5 years (e.g. from International Symposium on Paediatric Pain (ISPP), the Society of Paediatric Psychology Annual Conference (SPPAC), or the Paediatric Pain Conference (PPC) conferences). In addition, reference chaining will be implemented by reviewing the reference lists of all included papers to identify any significant articles that may have been overlooked.

## Data collection and analysis

### Selection of studies

One review author (SS) will initially screen titles and abstracts and eliminate those obviously not relevant to this review. A second review author (LH) will also screen 20% of all titles and abstracts and full text articles.

Ineligible studies will be excluded at this stage, and the authors will record the reason for rejection. When the title and abstract do not provide all the information concerning the criteria, full-text copies will be retrieved and screened. The review authors will retrieve full-text copies of all studies if either review author determines that the study possibly or definitely meets the inclusion criteria.

Disagreements between the two reviewers will be resolved by discussion, with the involvement of a third reviewer where agreement cannot be reached (LC or BMG). Multiple reports of the same study will be counted as a single study. The PRISMA template will be used to produce a flow chart showing details of studies included and excluded at each stage of the study selection process.

### Data extraction and management

A data extraction sheet will be created and piloted on a sample of three studies and then altered if required before full data extraction begins. The two review authors will then extract the data from the selected studies. Discrepancies will be resolved by discussion, with the involvement of a third reviewer where necessary. Authors will be contacted in order to obtain any necessary missing data. The following information will be extracted from the studies:

- Participant characteristics including demographic characteristics (e.g. age, sex)
- Disease-specific factors such as JIA type, longevity, pain variables, and treatment characteristics including types and duration of treatment
- Sibling and family data (e.g. ages, sex, socio-economic status)
- The geographic location of the study
- Inclusion/exclusion criteria for participation in study
- Relevant findings with respect to the level of family resilience and its impact on child QOL
- Classification of the resilience variables as predictors, moderators, mediators, or outcome variable

## Quality assessment of the included studies

The criteria to be used were developed to assess study quality for qualitative, quantitative, and mixed methods studies [10]. These criteria were established in response to an appeal by various published guidelines [11, 12, 13, 14, 15, 16, 17] to evaluate the scientific quality of the studies a researcher chooses for a review.

To apply this method, the researchers (SS & LH) will follow the guidelines by rating the quantitative studies on nine criteria, e.g. explicit scientific context and purpose, methods, measurement reliability and statistics, statistical power, internal validity, measurement validity, external validity, appropriate discussion, and contribution to knowledge. Accordingly, the qualitative studies will be rated on 11 criteria, e.g. explicit scientific context and purpose, methods, grounding in examples, the findings are integrated into a framework, owning one’s perspective, resonating with readers (the material is presented in such a way that readers, judge it to have represented accurately the subject matter or to have clarified or expanded their appreciation and understanding of it [12]), data is appropriate, credibility checks, situating the sample, appropriate discussion, and contribution to knowledge. To address studies using mixed methods (qualitative and quantitative designs), SS and LH will utilize all the presented criteria. Papers will be rated on a scale from low to high, where 1 represents low quality and 3 represents high quality [10]. Each paper will receive an average score over the various criteria. In both the qualitative and quantitative criteria decision making process, disagreements between SS and LH will be mediated by either BMG or LC.

## Data synthesis

Two review authors (SS and LH) will systematically and comprehensively assess the results of each study, explore the relationships in the data, and highlight the vital characteristics of the studies where relevant, such as important similarities or differences (for example, in study design, populations, interventions or other elements). Further, the review authors will assess the robustness of the synthesis based on the amount and quality of the evidence (i.e. the included studies), and the methods used to synthesize the evidence (i.e. how well the methods minimized bias). The results will be synthesized according to the Resilience Resources and Mechanisms in Paediatric Chronic Pain model [9].

The authors will further evaluate the quality of the studies using the Quality Assessment developed by Alderfer et al. [10] through which papers are rated on a scale of 1 (low—little or no evidence of fulfilling the guideline or doing poorly) to 3 (high—good evidence or high quality).

The researchers will be utilizing a text-based analysis in the form of a narrative synthesis. The researchers will refer to the Guidance on the Conduct of Narrative Synthesis in Systematic Reviews [18]. A narrative synthesis is appropriate in this context as the evidence is varied and underdeveloped. While a narrative synthesis can involve a manipulation of the data, we will not be taking that approach. Instead, the researchers will be focusing on a wide range of questions and summarizing the findings across the data. In addition, any measures that assess QOL and resilience in the studies will be included in the narrative synthesis.

## Subgroup analysis and investigation of heterogeneity

We expect different categories will be used by the included studies, including context of the chronic pain, types of resilience processes and mechanisms, and individual as opposed to family dynamics. Therefore, if sufficient data are available, we will undertake subgroup analysis based on the following:

1. Types of resilience: mechanism or resource
2. Individual approaches to resilience processes and mechanisms versus the family approaches to resilience processes and mechanisms

If appropriate, we will use subgroup analysis to categorize these studies and explore heterogeneity.

## Discussion

The proposed review will add to the literature in several ways. Research efforts to further the understanding of paediatric chronic pain experiences are usually focused on vulnerability factors for maladaptive coping in both children and their family, such as anxiety [19], depression [20], pain catastrophizing [21], and fear [22]. However, developmental psychology research has shown that many family units demonstrate substantial flexibility and resilience during negative effects or ongoing interference due to chronic illness [23].

Research exploring resilience within the adult population is rapidly accruing [24], and it supports positive emotional responses as a resilience resource [25]; however, the focus on resilience within the context of paediatric chronic pain is only recent [9]. In particular, there has been a scarcity of longitudinal approaches investigating how resilience can promote adjustment in children with a chronic illness [26]. Shifting our focus to protective factors and mechanisms of resilience in the context of paediatric chronic pain, specifically JIA, is a novel and promising pursuit that has the potential to optimize and inform future clinical practice and interventions. Recent literature validates family functioning as having a high level impact on child outcomes and demonstrates the positive effects of family-level processes and variables as resilience tools within paediatric chronic pain. However, this research is restricted to studies examining general family functioning and resilience instead of measures specific to chronic pain or JIA [27]. Though literature regarding family adjustment after a diagnosis of paediatric chronic illness is available [28], this work has not been conceptualized within the framework of family resilience theory [29]. Innovative research into the resilience of the family system, including parents and siblings, is important to understand the impact on the family as a whole, as they often alter roles and responsibilities to effectively communicate, manage emotions, and successfully work as a team to meet treatment demands [29].

As a first important step in this direction, this review will summarize the current evidence on how resilience affects the parents and siblings on an individual level while simultaneously concentrating on how resilience affects the family unit. This will be achieved through the application of the Resilience Resources and Mechanisms in Chronic Pain model developed by Cousins et al. [9] as the main analytical framework. This model will highlight and synthesize the findings in an accurate and constructive format. In addition, the use of the Resilience Resources and Mechanisms in Chronic Pain model will strengthen the value and quality of the summary as well as the rigorousness and thoroughness of the data.

Briefly, the model extrapolates the relationship between “resilience resources” and “resilience mechanisms” in the framework of paediatric chronic pain and has been successfully utilized within paediatric asthma and diabetes populations. The Resilience Resources model has identified several intricate systems that promote health, illness management, adaptability, and psychosocial functioning [30]. Potentially important additional constructs to be explored within this area include positive affect, post-traumatic growth, benefit finding, optimism, and self-regulation towards pain [9].

The authors of the Resilience Resources model acknowledge that both individual and family-based processes impact paediatric pain functioning and influence resilience [9]. Their model was adapted from Sturgeon and Zautra’s adult chronic pain risk-resilience model; however, Cousins et al. [9] adjusted it to include variables that are specific to, and have received support from, the paediatric chronic pain literature. Cousins et al. [9] stress that while research supports the negative effects of paediatric chronic pain, there is limited research available on positive adaptation and the extent to which resilience influences the family as a unit.

A better understanding of the role of reliance in family adaptation will facilitate the development of more effective treatment approaches and lay the foundation for more effective self-management in paediatric chronic pain.

## Additional file

Additional file 1: PRISMA-P 2015 Checklist. This checklist is approved for use with systematic review protocol submissions. (DOCX 32 kb)

## Abbreviations

BMG: Brian McGuire
BPS: British Psychological Society
ISPP: International Symposium on Pediatric Pain
JIA: Juvenile idiopathic arthritis
LC: Line Caes
LH: Lisa Hynes
PICO: Participants, Interventions, Comparisons, Outcome(s)
PPC: The Paediatric Pain Conference
PRISMA: Preferred Reporting Items for Systematic Reviews and Meta-Analyses
PROSPERO: Prospective Register of Systematic Reviews
QOL: Quality of life
SPPAC: Society of Pediatric Psychology Annual Conference
SS: Sophia Saetes
